# Comparison of actual and automated CT measurements of urinary stone size: a phantom study

**DOI:** 10.1007/s00240-025-01708-1

**Published:** 2025-04-11

**Authors:** Myeong Seong Yoon, Dong-Hyun Jang, Juncheol Lee, Jaehoon Jeong, Do Gwon Kim, Hyojin Lim, Dong Keon Lee, Jaehoon Oh

**Affiliations:** 1 Medical Imaging Processing Lab (MIP), Seoul, Republic of Korea; 2 Seoul National University Bundang Hospital, Seongnam-si, Republic of Korea; 3 Hanyang University, Seoul, Republic of Korea; 4 AIDOT Inc, Seoul, Republic of Korea; 5 Seoul National University College of Medicine, Seoul, Republic of Korea

**Keywords:** Urinary stone, Stone phantom, Computed tomography, Stone size

## Abstract

Urinary stone size is key in determining treatment. Although computed tomography (CT) scans are widely used for diagnosing urinary stones, measurements of stone size obtained from CT images may be inaccurate compared to actual size. Twenty-four urinary stone phantoms were 3D printed at three densities (100, 1000, and 3000 Hounsfield units [HU]) and eight sizes. CT images of the phantoms were taken. Nineteen radiologists and 33 emergency physicians from two institutions measured stone sizes on CT images using mediastinum and bone settings. An automated algorithm segmented regions of interest and estimated stone size using pixel HUs. Mean absolute error (MAE) was assessed for the accuracy of each measurement method against known phantom sizes. For the mediastinum setting, MAEs for 100, 1000, and 3000 HU stone phantoms were 1.05 mm ± 0.06, 1.01 mm ± 0.06, and 2.38 mm ± 0.17, respectively. For the bone setting, MAEs were 0.98 mm ± 0.07, 0.55 mm ± 0.10, and 1.91 mm ± 0.06, respectively. For automated measurements, MAEs were 1.16 mm, 0.21 mm, and 2.10 mm, respectively. Participant-to-participant variability was observed across all measurement settings, regardless of the stone density or window used. For stone size measurements on CT images, the bone setting provided more accurate results than the mediastinum setting. Automated measurement methods, which estimate stone size by outlining its edges, were more accurate than manual measurements for 1000 HU stones, the most common stone density. However, for stones with densities above or below 1000 HU, the accuracy of the automated method may decrease.

**Supplementary Information:**

The online version contains supplementary material available at 10.1007/s00240-025-01708-1.

## Introduction

Urinary stone disease is a common condition, with a prevalence as high as 10% [1]. It occurs when kidney deposits that are not properly excreted from the kidneys crystallise in the urinary tract, which may lead to obstruction [2]. Treatment urgency depends on the severity of complications from obstruction [3]. As urinary stone size is a primary determining factor in assessing obstruction risk, it influences treatment decisions.

Computed tomography (CT) shows satisfactory sensitivity and specificity for diagnosing urinary stone disease [3, 4]. It identifies stone location and size, aiding in treatment planning. However, the measurement of stone size on CT scans can be inaccurate compared with that of the actual size [5, 6]. Variability in stone attenuation patterns, based on composition, can show inconsistent results across measurement methods and window settings. Furthermore, CT artefacts, such as beam-hardening in high-density stones, may further reduce measurement accuracy.

Diagnostic studies are exploring algorithms to assist in image interpretation for urinary stone disease [7–9]. However, evidence regarding the accuracy of CT stone size measurements compared with the actual size is limited.

The primary objective of this phantom study was to assess the accuracy of urinary size estimation based on CT window settings, specifically comparing the accuracy of measurements taken with mediastinum and bone window settings. Additionally, it aimed to determine the accuracy of an automated measurement model for measuring urinary stone size compared to the actual stone size.

## Materials and methods

### Phantom product

**Fig. 1:**
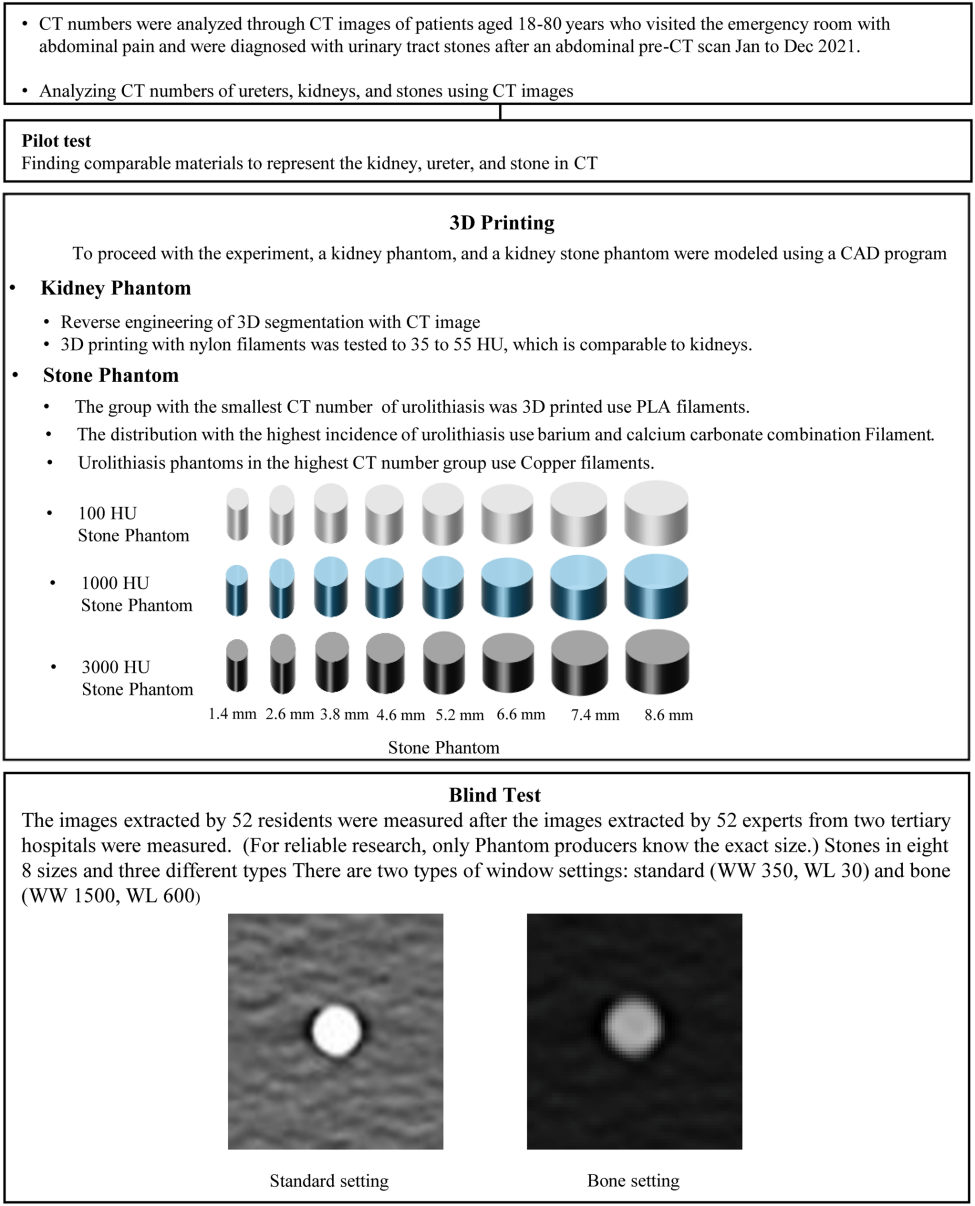
Process flow for creating and evaluating urinary stone phantom

Given that urinary stones have varying attenuation based on composition leading to different degrees of artefact in CT images, stone phantoms were created with three different Hounsfield units (HUs): approximately 100, 1000, and 3000. The production process and size measurement procedure for the urinary stone phantoms are summarised in Fig. 1.

All the urinary stone phantoms were produced using a 3D printer (Ultimaker S3, Netherlands) combined with commercial filaments. A pilot test was conducted to evaluate approximately 10 commercial filaments for their suitability in fabricating urinary stone phantoms, focusing on materials capable of achieving stable and reproducible attenuation values that represent the range of densities observed in human urinary stones. Different types of commercial filaments were selected, and 3D printed as a 20 × 20 × 50 mm cylinder. The fabricated cylindrical models were placed in the centre of a plastic box filled with water (dimensions: 36 × 25 × 15 cm^3^) for CT scans to measure the densities of each candidate material. The density of the water inside the box was also confirmed to be within 0 ± 7 HU to confirm density measurement accuracy.

Based on pilot test results, urinary stone phantoms with three distinct densities were produced using a combination of commercial filaments to analyse measurement error variations associated with stone phantom densities. Attenuation values of 100 HU, 1000 HU, and 3000 HU were selected to evaluate measurement accuracy across a broad range of densities, including extreme values, to better understand potential artefacts in varying clinical scenarios. These attenuation values were chosen based on a review of several reports on urinary stone densities, which indicated that stone densities typically range from the low 100s to the high 2000s, depending on composition [10, 11]. Stone phantoms were produced in eight sizes ranging from 1 to 8 mm for each density. Polylactic acid (126 HU) was selected for 100 HU stone phantoms in the lowest-density group, and copper (3071 HU) for 3000 HU stone phantoms at the highest density. Owing to the lack of commercially available filaments matching the required density for 1000 HU stone phantoms, a 1000 HU stone phantom was fabricated using a combination of barium and calcium carbonate. For the stone phantoms, attenuation values were calculated as the mean HU across the entire measured region.

Using 3D modelling software (AVIEW-Modeler, Coreline Soft, Korea), urinary tract phantoms were modelled and 3D-printed in eight sizes, each with three densities determined via a pilot study. Parameters such as the optimal nozzle temperature, bed temperature, nozzle size, output speed, and layer height for each material were optimised using a slicing program (Ultimaker Cura 5.2.1, Netherlands) to improve output quality. The parameters were subsequently transferred to a 3D printer to print stone phantoms. The printed stone phantoms were inspected for errors using digital callipers (CD-6”ASX, Mitutoyo, Japan).

The fabricated stone phantoms were inserted into a latex drainage tube with size and density (10–20 HU) similar to those of a human ureter and fixed at the centre of a plastic box filled with water. For CT imaging, 24 stone phantoms (eight different sizes for each of the three densities) were randomly placed across six latex drainage tubes, with four stone phantoms per tube. Sufficient spacing was maintained between stone phantoms to prevent interference during scanning. The water bath was filled with distilled water to complete the urolithiasis phantoms (Supplementary Fig. 1).

### Computed tomography scan parameters

The CT scans of urinary stone phantoms were performed using a 128-channel CT machine (CT-WS-21 A, HITACHI, Japan). Scan parameters typically used for clinical diagnosis of urolithiasis via abdominal CT were applied, including Auto Exposure Control (AEC) mode with an average of 125 mAs, a tube voltage of 120 kVp, and a slice thickness of 1.0 mm.

### Size measurements of urinary stone phantoms

The CT images were acquired in DICOM (Digital Imaging and Communications in Medicine) format from the Picture Archiving and Communication System for measurement. To compare stone size measurements on CT images with the actual sizes, 19 radiologists and 33 emergency physicians from two tertiary academic hospitals located in Seoul and Gyeonggi-do, Republic of Korea, were recruited. Participants were blinded to the exact size and density of the stone phantoms. During measurement, the mediastinum setting (window width 350 HU, window level 30 HU) and bone setting (window width 1500 HU, window level 600 HU) were used to analyse the effect of window setting on stone size measurement [12]. Only axial images were utilized for the measurement process, as the study’s design defined the phantom’s actual size based on the diameter of the cylindrical phantom. Axial images were deemed the most relevant plane for direct comparison between the actual and measured sizes.

There was no unified protocol for manual stone size measurements beyond the standardized window settings, and participants were allowed to zoom into the images at their discretion. Each participant was provided with a single static axial image for each phantom, and scrolling through image stacks or accessing coronal and sagittal views was not applicable. Each of the 24 stone phantoms (eight per density group) was measured once by each participant. CT images of the stone phantoms were presented in a randomised order.

### Automated model for urinary stone size measurement

**Fig. 2:**
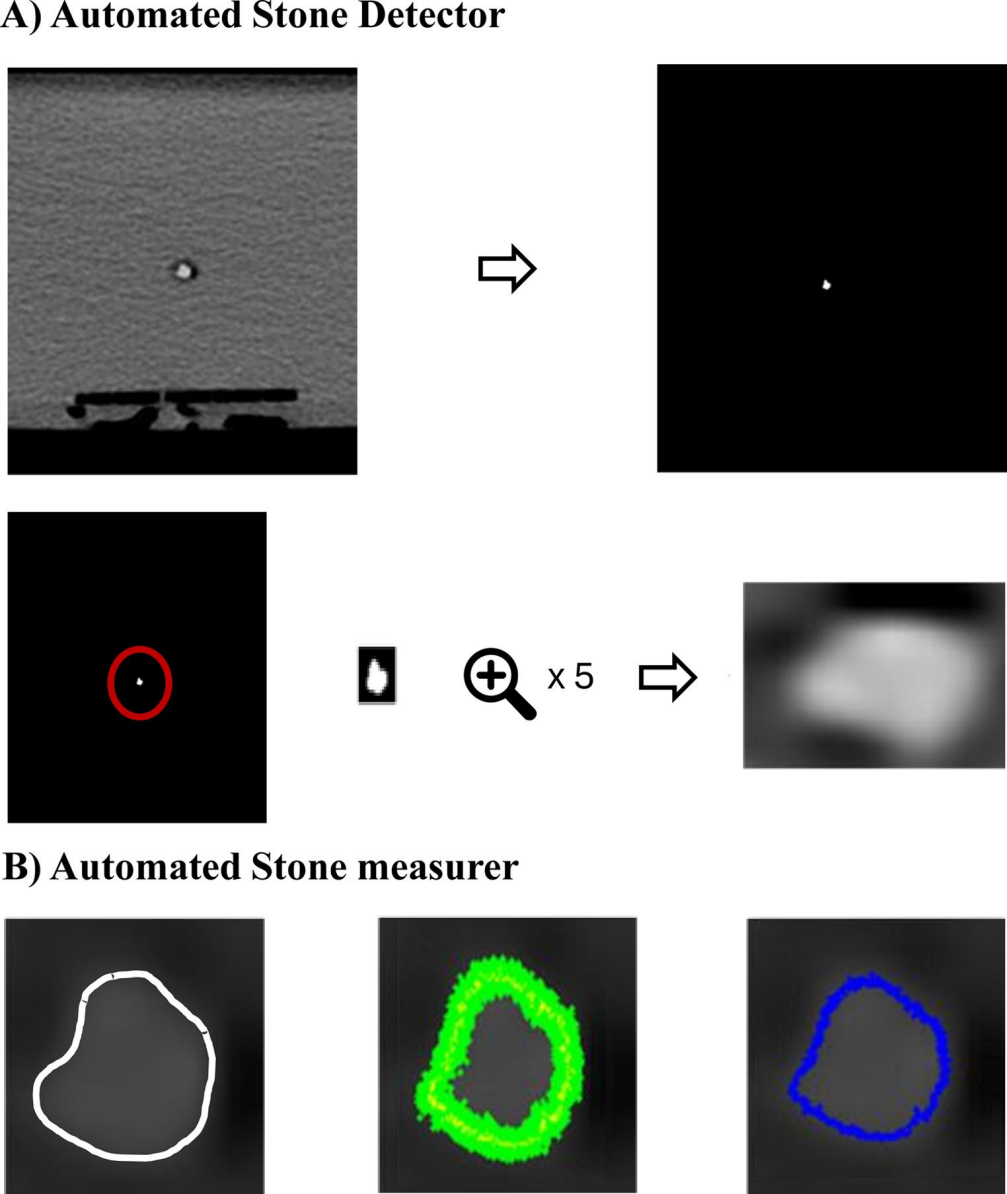
Proposed steps for automated stone size measurement. (**A**) The original CT image of a stone phantom is changed to the bone setting. The region of interest (ROI) is cropped and magnified. (**B**) A first-order Haar wavelet is applied to detect edges, followed by the application of a bone window (500, 1600) to estimate edge corners

An automated stone size measurement algorithm was proposed to determine the size of stone phantoms from CT images, which was divided into two main modules (Fig. 2).

**Fig. 3:**
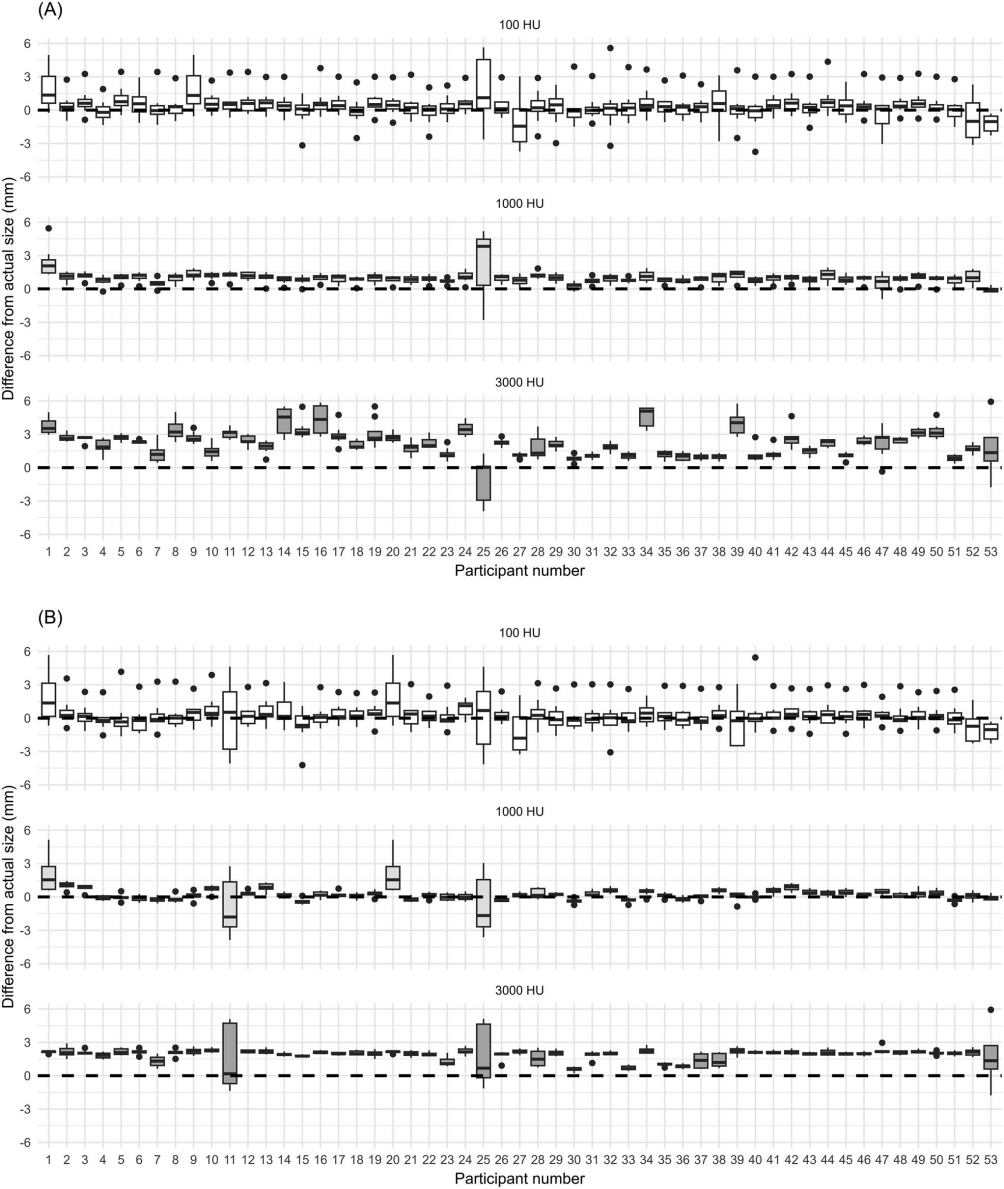
Distribution of measurement differences from actual stone size by density. Numbers 1 to 52 represent individual participants, and number 53 corresponds to the automated measurement. (**A**) Measurements by 52 participants and the automated result under the mediastinum setting. (**B**) Measurements by 52 participants and the automated result under the bone setting

The Automated Stone Detector identified a set of stones within a CT image by binarizing the input series based on the HU of each pixel. Stone location candidates were detected based on the HU, building a three-dimensional map. Thereafter, connected component labelling was applied to the binarized image to isolate potential stones and distinguish them from other structures. Non-stone voxel areas detected for each candidate were cropped, and the remaining area was designated as the region of interest.

The Automated Stone Measurer module analysed each slice’s input region of interest. An imaginary line was drawn from the centre of the stone to the outermost edge of the image. Subsequently, a 1D Haar filter, a first-order differential filter, was applied to the points on the line to detect the minima, marking the outline of the stone. The Euclidean distance in voxel units between detected outline points was calculated. The maximum distance was reported as the size of the measured stone.

### Statistical analyses

To evaluate each measurement method’s alignment with the actual stone size, the mean absolute error (MAE) was calculated. A Student’s *t*-test determined whether statistical significance in actual size measurement accuracy among the different methods. As CT scans can induce varying degrees of artefacts based on subject density, affecting measurement accuracy (Supplementary Fig. 2), all analyses were performed separately according to stone phantom density.

To evaluate the accuracy of the manual measurements in both window settings and compare it with the automated measurement, absolute errors for each participant’s measurements were calculated. Additionally, to identify any method-specific tendencies to overestimate or underestimate the actual size, the distribution of differences between the measurements and the actual size was compared using stone phantoms and their densities.

To assess participant-to-participant variability in manual measurements, absolute errors were calculated for each stone phantom. For each participant, mean ± standard deviation, minimum, and maximum values of absolute errors were calculated across all measured stones. These results were summarised according to stone density (100, 1000, and 3000 HU) and measurement setting (mediastinum and bone).

All statistical analyses were performed using the R statistical software (version 4.3.1; R Foundation for Statistical Computing, Vienna, Austria). Statistical significance was defined as *p* < 0.05.

## Results

The absolute errors between the actual sizes and measurements obtained from manual measurements using mediastinum and bone settings, and the automated measurement model, are presented in Fig. 2. Compared with manual measurements in the mediastinum setting, the automated model exhibited a lower MAE for 10 participants (19.2%) with 100 HU stones, all 52 participants (100%) with 1000 HU stones, and 29 participants (55.8%) with 3000 HU stones. In comparison to manual measurements in the bone setting, the automated model exhibited a lower MAE for seven participants (13.5%) with 100 HU stone phantoms, 45 participants (86.5%) with 1000 HU stone phantoms, and 20 participants (38.5%) with 3000 HU stone phantoms. Among manual measurement results for each participant across different window settings, the bone setting demonstrated superior accuracy compared with the mediastinum setting, with 36 participants (69.2%) for the 100 HU stone phantoms, 48 participants (92.3%) for the 1000 HU stone phantoms, and 37 participants (71.2%) for the 3000 HU stone phantoms. The MAEs for each measurement method are listed in Table 1. Of the three methods, manual measurements using the mediastinum setting exhibited the highest MAE across all densities. For the 100 HU and 3000 HU stone phantoms, manual measurements using the bone setting yielded the most accurate results. For the 1000 HU stone phantom, the automated measurement method demonstrated the highest accuracy.

**Table 1:** Mean absolute error of stone phantoms by different measurement methods

Density of stone phantom	Mediastinum setting	Bone setting	Automated measurement	*p*_a_	*p*_b_	*p*_c_
100 HU	1.05 ± 0.06	0.98 ± 0.07	1.16	0.255	0.06	0.009
1,000 HU	1.01 ± 0.06	0.55 ± 0.10	0.21	< 0.001	< 0.001	0.001
3,000 HU	2.38 ± 0.17	1.91 ± 0.06	2.10	0.004	< 0.001	0.002

HU, Hounsfield unit

Mean absolute error for the mediastinum setting and bone setting measurements were calculated from 52 participants and expressed as mean ± sd

p_a_, p value for comparison between manual measurement in mediastinum setting and manual measurement in bone setting; p_b_, p value for comparison between manual measurement in mediastinum setting and automated measurement, p_c_, p value for comparison between manual measurement in bone setting and automated measurement

**Table 2:** Participant-to-participant variability in error in stone size measurements across mediastinum and bone settings

Density of stone phantom	Actual size of the stone phantom (mm)	Error in mediastinum setting (mm)		Error in bone setting (mm)		Error in automated measurement (mm)
		Mean ± sd	Range (min–max)	Mean ± sd	Range (min–max)	
100 HU	1.4	3.1 ± 0.8	0.7–5.6	2.9 ± 0.9	1.1–5.7	0.4
	2.6	1.3 ± 1.2	0.1–6.1	1.1 ± 1.0	0.0–4.6	0.3
	3.8	0.5 ± 0.9	0.0–4.7	0.5 ± 0.5	0.0–2.9	0.8
	4.6	0.3 ± 0.3	0.0–1.8	0.5 ± 0.6	0.0–2.4	1.8
	6.6	0.9 ± 1.1	0.0–3.8	0.6 ± 1.0	0.0–4.2	2.3
	6.6	1.0 ± 0.6	0.2–3.7	1.2 ± 0.5	0.1–3.3	2.1
	7.4	0.6 ± 0.6	0.0–3.1	0.6 ± 1.0	0.0–4.2	0.6
	8.6	0.7 ± 0.7	0.1–4.5	0.5 ± 0.5	0.0–2.5	1.3
1000 HU	1.4	0.9 ± 0.8	0.2–5.4	0.7 ± 1.0	0.0–5.1	0.2
	2.6	0.4 ± 0.7	0.0–4.3	0.6 ± 1.3	0.0–6.2	0.3
	3.8	1.0 ± 0.7	0.1–5.2	0.8 ± 1.4	0.0–7.2	0.1
	4.6	1.1 ± 0.3	0.3–2.4	0.5 ± 0.6	0.0–2.8	0.1
	5.2	1.0 ± 0.6	0.4–5.1	0.4 ± 0.6	0.0–3.1	0.4
	6.6	1.2 ± 0.3	0.5–2.8	0.4 ± 0.3	0.0–1.2	0.3
	7.4	1.2 ± 0.5	0.4–3.6	0.5 ± 0.7	0.0–3.9	0.3
	8.6	1.2 ± 0.4	0.3–1.9	0.5 ± 0.5	0.0–2.7	0.0
3000 HU	1.4	2.5 ± 1.2	0.7–8.0	1.9 ± 0.7	0.6–5.1	1.4
	2.6	2.1 ± 1.1	0.3–4.9	2.0 ± 0.7	0.4–4.6	2.7
	3.8	2.5 ± 1.6	0.4–6.1	1.7 ± 0.6	0.0–2.4	1.8
	4.6	2.3 ± 1.5	0.1–5.9	2.0 ± 0.7	0.7–5.1	2.7
	5.2	2.2 ± 1.4	0.1–6.4	1.8 ± 0.5	0.5–2.3	5.9
	6.6	2.6 ± 1.8	1.1–8.9	1.9 ± 0.4	0.8–2.9	0.2
	7.4	2.3 ± 1.6	0.1–8.0	2.0 ± 0.6	0.3–2.7	0.7
	8.6	2.5 ± 2.1	0.8–10.0	2.0 ± 0.7	0.1–3.0	1.3

HU, Hounsfield unit

Range indicates the minimum and maximum errors observed in each setting

The distribution of differences between the measured values from each measurement method and the actual size for each stone phantom density is shown in Fig. 3. For 100 HU stone phantoms, the automated measurement underestimated the actual size compared to both the actual size and manual measurements. For 1000 HU stone phantoms, manual measurements in the mediastinum setting overestimated the size compared with the actual size. For the 3000 HU stone phantoms, manual measurements in both window settings overestimated the size compared with the actual size, with the mediastinum setting showing a greater overestimation than the bone setting.

The participant-to-participant variability for each stone density across measurement settings is summarised in Table 2. While the bone setting demonstrated lower MAE overall compared to the mediastinum setting, significant variability was observed in both settings across all stone densities. Notably, for 1000 HU stone phantoms, the mean absolute error was relatively low; however, the range of errors (min–max) remained substantial, indicating the absence of a clear trend in measurement consistency.

## Discussion

The study results demonstrated that accuracy in estimating urinary stone size varies with window settings for different HU values in CT images. Bone setting measurements were more accurate than soft tissue settings. The automated measurement model performed better or comparably to manual measurements in the mediastinum setting. Compared with manual measurements in the bone setting, the automated model exhibited inferior performance for 100 and 3000 HU stone phantoms and superior performance for 1000 HU stone phantoms.

Guidelines state that stone size is crucial for predicting spontaneous expulsion or expulsion with medical treatment is possible [3, 13, 14]. Most urinary stones with sudden-onset symptoms are < 1–2 cm in size, and small measurement differences can affect treatment decisions. Accurate non-invasive estimation of stone size in millimetres is essential for treatment planning as actual size can only be confirmed post-expulsion. Abdominopelvic CT is commonly used for diagnosing urinary stones and determining appropriate treatment strategies [3, 4]. Variations in HU depending on stone composition and CT artefacts can affect measurement accuracy [15]. However, most guidelines lack specific instructions for accurately measuring stone size on CT scans.

Inconsistent results have been reported regarding the accuracy of urinary stone size measurements on CT images compared with the actual stone size. Kishore et al. found that CT-measured distal ureter stones were generally larger than actual spontaneously expelled stones, with a low correlation coefficient [5]. Therefore, this study concluded that CT stone size measurements may be inaccurate and should be interpreted with caution. Reimer et al. conducted a phantom study that simulated the kidney and kidney stones within it, reporting that manual measurements often overestimated stone size compared to the actual size, yet had a good correlation [16]. The study also noted that CT image reconstruction algorithms and kernels could impact measurement accuracy; however, radiation dose and denoising had minimal effects. The inconsistencies observed in previous studies may be attributed to the lack of a standardised measurement method. Our study similarly showed that measurement errors varied by stone density, measurer, and CT window setting.

High-density objects such as stones often produce artefacts on CT [15], leading to size overestimation. Moreover, artefact appearance and size measurements can vary by window setting, impacting accuracy. Umbach et al. analysed the correlation between actual stone size and CT measurements using different slice thicknesses and window settings for uric acid stones collected from a patient and phantom stones [17]. Their results showed that CT measurements generally correlated well with actual size, especially with thinner slices and the bone window setting. This study found that measurement correlation with the actual size varied depending on CT slice thickness and window setting. The study reported that measurements made in the bone window setting aligned more closely with the actual stone size than those made in other window settings. Reimer et al. performed a phantom study comparing urinary stone sizes in various CT window settings to their actual sizes [12]. Reimer et al. found that the accuracy of measurement varied by window setting, with the bone setting providing the most accurate values. Although previous studies have reported that the accuracy of CT-based urinary stone size estimates can vary by measurement method, limited evidence suggests that inaccuracy may also depending on stone density.

We hypothesised that the density of stone phantoms, a key factor in CT artefact formation in CT images, impacts size measurement accuracy. We compared stone phantom sizes measured by clinicians at three densities using bone and mediastinum window settings. Results showed that numerous clinicians overestimated stone phantom sizes relative, especially with higher densities and in the mediastinum setting. In diagnosing urinary stone disease, where millimetre-scale differences in measurements influence treatment plans, these findings suggest considering stone density and window setting errors when estimating actual stone size on CT.

Several studies have evaluated urinary stone size using commercially available automated tools for calcified lesions or volume on CT, comparing results with manual measurements [6, 18]. However, evidence is limited on the accuracy of automated measurement methods that use stone margins to estimate actual stone size, and performance difference compared with manual measurements. This study developed a model to automatically extract stone margins visible on bone-setting images to estimate stone size and compared these automated measurements with the actual stone phantom size and manual measurements. Analysis results showed that for 1000 HU stone phantoms, the most common stone density, automated measurements were more accurate than manual ones. However, for 100 and 3000 HU stone phantoms, automated measurements overestimated size compared to manual measurements. These findings suggested that in cases of radiolucent stones or considerably large artefacts, manual measurement may account for these factors when estimating stone margins, whereas automated methods outline the stone directly without considering these factors, potentially causing overestimation.

Manual measurements in imaging inherently suffer from variability between individuals, which can compromise reliability and reproducibility [19]. In the analysis of medical images, particularly in size measurement, variability among individuals performing the measurements highlights a fundamental limitation in achieving objective evaluation. Such variability is particularly concerning when small deviations in measurements carry significant clinical implications, such as in urinary stone size estimation and related treatment decisions. Previous studies have suggested that stone size and location, as evaluated on CT, are key predictors of spontaneous ureteral stone passage, even when the actual stone size is not precisely known [20]. However, consistent measurements remain crucial for accurate predictions [19]. Although our study did not provide a standardised protocol beyond the window settings for measuring stone size, the results illustrate the extent of variability that can arise due to differences in interpretation or environment, as may occur in real-world clinical settings. These findings suggest the limitations of manual methods and further highlight the potential benefits of automated measurement techniques in mitigating variability, thereby improving consistency and reliability in clinical practice.

This phantom study focused on comparing the accuracy of manual and automated measurement methods under different window settings, rather than detecting stones in actual CT images or distinguishing them from surrounding structures. As such, the study was conducted under controlled conditions, and the process of differentiating stones from other high-attenuation lesions was not included. The automated measurement method proposed in this study showed high accuracy for 1000 HU stone phantoms, which represent the most common stone density. However, additional studies are needed to determine whether our findings can be replicated in clinical settings, where CT scans often involve more complex anatomical structures. Furthermore, challenges remain in distinguishing stones from surrounding structures or other high-attenuation lesions in actual patient CT images. Based on a previous study about urinary tract segmentation on CT images, we believe that, by determining whether high-attenuation lesions are located within the segmented urinary tract, it may be possible to distinguish stones from other structures [21]. Additionally, morphology-based methods to differentiate stones from other high-attenuation lesions could serve as a supplementary approach for stone classification [22, 23].

The present study had several limitations. First, as a phantom study, the influence of surrounding body structures on measurements was excluded. Second, stone phantoms were fabricated in a cylindrical shape, which appeared round on axial CT images. This differs from the oval or irregular shapes of actual urinary stones, which may have less distinct edges and greater variability in grayscale artefacts. Additionally, although the cylindrical phantoms were positioned horizontally and perpendicular to the CT scan plane to the best of our ability, slight angulation cannot be completely excluded. Such angulation could have introduced minor variations in the cross-sectional measurements. Third, the attenuation values of 100 HU and 3000 HU for the stone phantoms, although chosen to evaluate measurement accuracy at the extremes of the reported density range, may not fully represent the typical densities encountered in clinical practice. While the 1000 HU phantom aligns closely with the density of common urinary stones, the use of extreme densities might limit the generalisability of the findings to more typical clinical scenarios.

## Conclusions

For stone size measurements on CT images, the bone setting provided more accurate results than the mediastinum setting. Automated measurement methods, which estimate stone size by outlining its edges, were more accurate than manual measurements for 1000 HU stones, the most common stone density. However, for stones with densities above or below 1000 HU, the accuracy of the automated method may decrease.

## Electronic supplementary material

Below is the link to the electronic supplementary material.

Supplementary Material 1

## Data Availability

No datasets were generated or analysed during the current study.
