# Supplementary material for: Comparison of actual and automated CT measurements of urinary stone size: a phantom study
Source: Urolithiasis. 2025 Apr 11;53(1):71. doi: 10.1007/s00240-025-01708-1 (PMC11991932; doi:10.1007/s00240-025-01708-1)
Supplement: Supplementary file 1 — Supplementary Material 1 [file 240_2025_1708_MOESM1_ESM.docx]

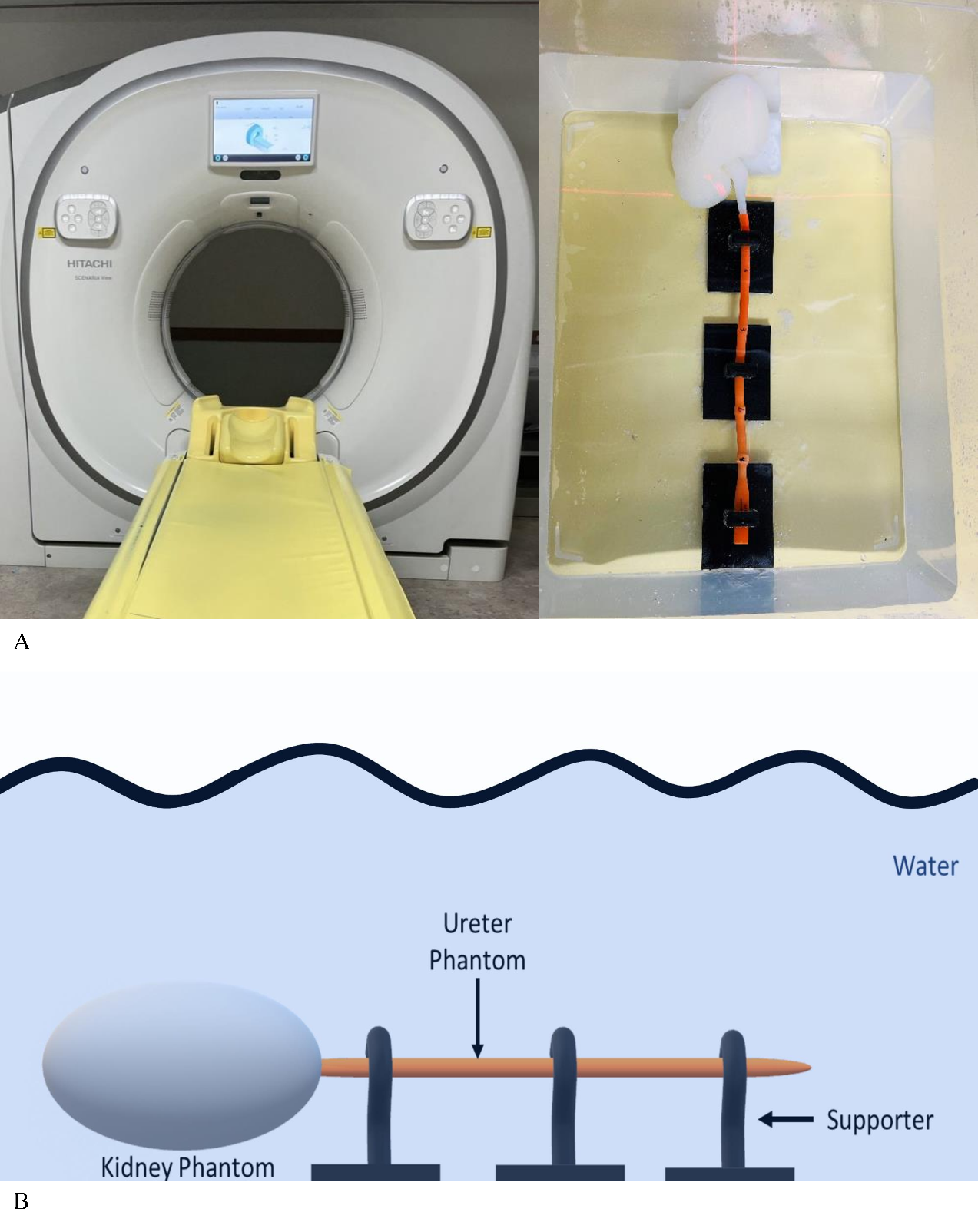


Supplementary Fig. 1. **Stone Phantoms Prepared for CT Scanning.** (A) Stone phantoms with varying CT numbers are randomly arranged within latex drainage tubes to simulate the ureter for imaging purposes. (B) Schematic diagram of the setup for CT scanning.


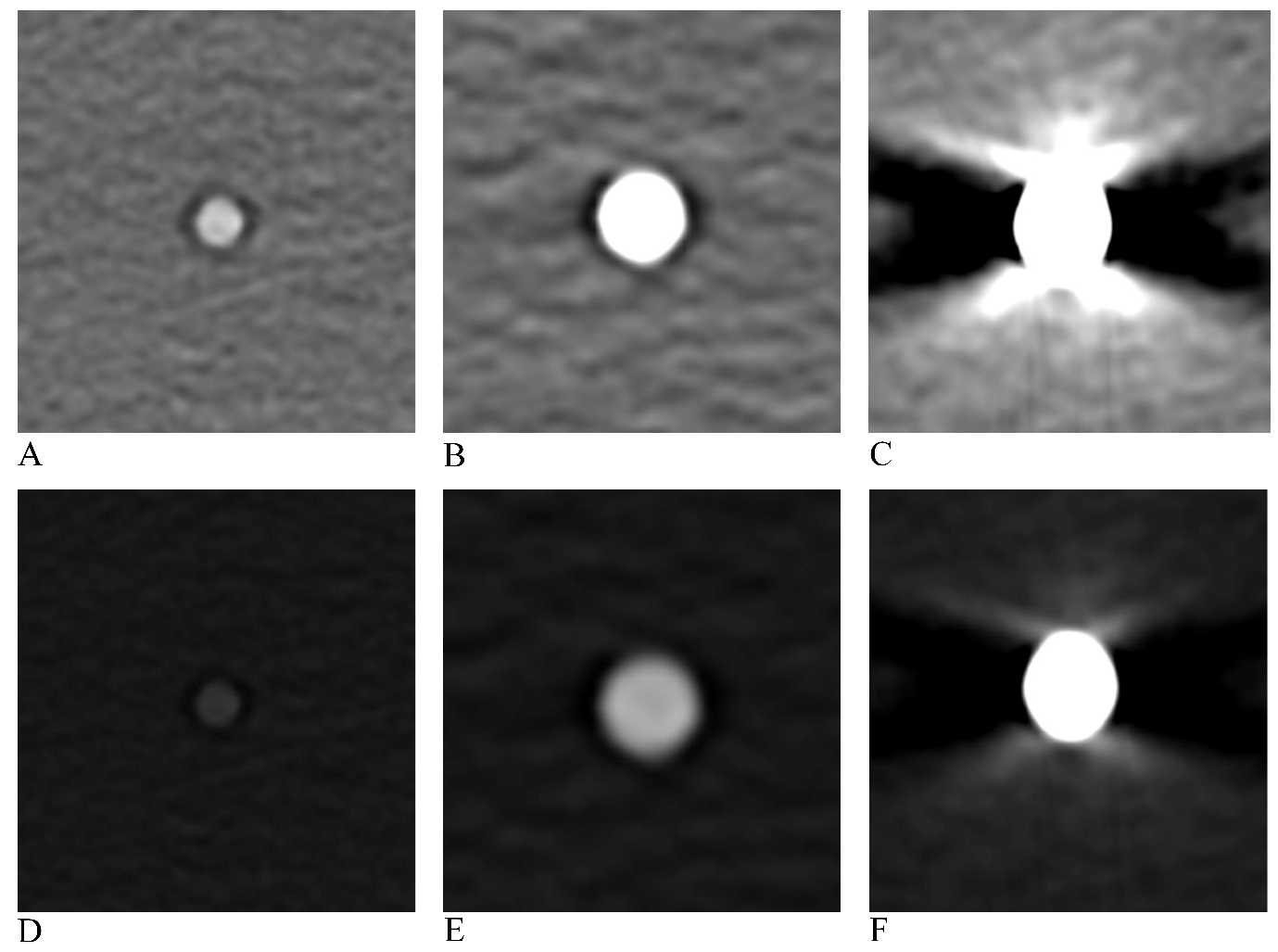


Supplementary Fig. 2. **Representative CT Scan Images of Urinary Stone Phantoms for Size Measurement.** (A) and (D) show images of a 100 Hounsfield Unit stone phantom with mediastinum and bone settings, respectively. (B) and (E) display a 1000 Hounsfield Unit stone phantom with mediastinum and bone settings. (C) and (F) depict a 3000 Hounsfield Unit stone phantom with mediastinum and bone settings.
